# Possible role of NO/NMDA pathway in the autistic-like behaviors induced by maternal separation stress in mice

**DOI:** 10.1371/journal.pone.0292631

**Published:** 2023-10-10

**Authors:** Fatemeh Khaledi, Hossein Tahmasebi Dehkordi, Elham Zarean, Mehrdad Shahrani, Hossein Amini-Khoei

**Affiliations:** Medical Plants Research Center, Basic Health Sciences Institute, Shahrekord University of Medical Sciences, Shahrekord, Iran; Belgrade University Faculty of Medicine, SERBIA

## Abstract

Autism spectrum disorder (ASD) is a complex neurodevelopmental disorder. Maternal separation (MS) stress is an established model of early-life stress associated with autistic-like behaviors. Altered glutamatergic and nitrergic neurotransmissions may contribute to the pathophysiology of ASD. However, the specific mechanisms underlying these alterations and their relationship to MS-induced autistic-like behaviors remain unclear. Addressing this knowledge gap, this study aims to elucidate the involvement of the nitric oxide (NO)/ N-methyl-D-aspartate (NMDA) pathway in MS-induced autistic-like behaviors in mice. This knowledge has the potential to guide future research, potentially leading to the development of targeted interventions or treatments aimed at modulating the NO/NMDA pathway to ameliorate ASD symptoms. Ninety male Naval Medical Research Institute (NMRI) mice were assigned to six groups (n = 15) comprising a control group (treated with saline) and five groups subjected to MS and treated with saline, ketamine, NMDA, L-NAME, and L-arginine. Behavioral tests were conducted, including the three-chamber test, shuttle box, elevated plus-maze, and marble burying test. Gene expression of iNOS, nNOS, and NMDA-R subunits (NR2A and NR2B), along with nitrite levels, was evaluated in the hippocampus. The findings demonstrated that MS induced autistic-like behaviors, accompanied by increased gene expression of iNOS, nNOS, NR2B, NR2A, and elevated nitrite levels in the hippocampus. Modulation of the NO/NMDA pathway with activators and inhibitors altered the effects of MS. These results suggest that the NO/NMDA pathway plays a role in mediating the negative effects of MS and potentially contributes to the development of autistic-like behaviors in maternally separated mice.

## Introduction

Autism spectrum disorder (ASD) is a complicated neurodevelopmental disorder that is typified by deficits in social communication, repetitive and restricted behaviors, as well as sensory abnormalities [1]. The etiology of ASD is complex and multifactorial, involving both genetic and environmental factors [2]. Maternal separation (MS) is a commonly used animal model of early-life stress (ELS) [3]. This model has been used extensively to investigate the impact of ELS on neurodevelopment, social communication, and behaviors [4]. There is growing evidence that ELS including MS is associated with the development of ASD [5, 6].

One of the prominent neurobiological changes associated with MS is altered glutamatergic neurotransmission, which has been suggested to contribute to the development of ASD [7, 8]. Glutamate is the primary stimulatory neurotransmitter in the central nervous system (CNS). It regulates several physiological processes, including learning and memory, neurodevelopment, and neuronal plasticity [9, 10]. It also acts on various ionotropic and metabotropic receptors, including N-methyl-D-aspartate (NMDA) receptors [11]. NMDA receptors (NMDA-Rs) are glutamate-gated ion channels that are widely expressed throughout the brain and are essential for normal synaptic transmission and plasticity [12, 13]. The NR2 subunit of NMDA-R is essential for NMDAR function and has four different subtypes (NR2A, NR2B, NR2C, and NR2D) encoded by separate genes. NR2A and NR2B subunits are the most abundant in the brain and have a major role in synaptic plasticity [14]. Several lines of evidence suggest that NMDA-Rs may contribute to the pathophysiology of ASD [15]. For instance, animal models of ASD have also shown abnormalities in NMDAR-mediated neurotransmission and synaptic plasticity [16, 17]. Previous studies have determined that dysfunction in the NMDA receptors and imbalances in neuronal excitatory and inhibitory synapses is involved in the pathophysiology of ASD. Indicating that dysfunction at excitatory synapses is associated with ASD [18]. In this regards it has been determined that the NMDA-R agonist, cycloserine, could ameliorate autistic symptoms in individuals with ASD [19]. On the contrary, some evidence demonstrated that elevated NMDA-R function is also involved in ASDs. In this concept, it has been shown that Memantine, an NMDA-R antagonist, improved ASD-related symptoms [20, 21].

Nitric oxide (NO) has a major effect on the balance of glutamatergic neurotransmission [22]. This molecule is produced through the conversion of L-arginine (L-arg) by the enzyme nitric oxide synthase (NOS), which is present in three different forms: neuronal NOS (nNOS), endothelial NOS (eNOS), and inducible NOS (iNOS).[23, 24]. nNOS and eNOS are constitutively expressed and produce NO under normal physiological conditions, whereas iNOS is induced by various stimuli such as inflammatory cytokines and lipopolysaccharides and produces large amounts of NO [25]. NO regulates synaptic plasticity and is involved in the control of various physiological functions, such as neurotransmission. Additionally, it plays a critical part in the regulation of NMDA-R [26]. Besides, NO selectively regulates the S-nitrosylation (SNO) of proteins that control glutamate transport and metabolism via the nNOS-dependent pathway. The dysregulation of this process may contribute to the pathogenesis of various neurological disorders, including ASD [27]. Previous studies have demonstrated that ELS such as MS increased the level of nitrite, a final product of NO in the brain [28, 29]. It has been shown that there is a direct association between levels of nitrite in the brain with neurological and neurodevelopmental disorders [30–33]. Pathogenesis of ASD is also assumed to involve a variety of biological flows activated by NO. The excessive NO levels lead to the production of reactive oxygen/nitrogen species (ROS/RNS) leading to mitochondrial dysfunction and neuroinflammation as well as oxidative stress which totally deteriorates autistic behaviors [34]. The effects of NO in ASD can either be neuroprotective or neurotoxic, and this is determined by the antioxidant capacity and the levels of ROS/RNS associated with oxidative stress [35]. Indicating that imbalance in the NO levels is involved in the pathophysiology of ASD, and can worsen the symptoms of this disease [36]. Furthermore, various molecular components including iNOS, interferon-gamma (IFN-γ), toll-like receptors 2, 3, and 4 (TLR2, TLR3, TLR4), nuclear factor-kappa B (NF-κB), and Adenosine A2A receptor (A2AR) signaling pathway intricately contribute to the neuroimmune dysfunction, exerting a profound influence on the pathogenesis of ASD. The dysregulation of these key elements disrupts the delicate balance of immune responses within the CNS, leading to aberrant neuroinflammation and neurodevelopmental processes underlying ASD [37–39].

In this study, we aimed to investigate the possible involvement of the NO/NMDA pathway in the autistic-like behaviors in a mouse model of MS stress focusing on the probable alterations in the gene expression of iNOS, nNOS, NR2B, and NR2A as well as levels of nitrite in the hippocampus.

## Materials and methods

### Ethics

All experiments described in this study were conducted in accordance with the Guide for the Care and Use of Laboratory Animals (8th edition, National Academies Press) set forth by the National Institutes of Health (NIH) and were approved by the Ethics Committee of the Shahrekord University of Medical Sciences (Ethics code: IR.SKUMS.REC.1399.256). All efforts were made to minimize animal suffering and to reduce the number of animals used.

### Study design

In this study, pregnant Naval Medical Research Institute (NMRI) mice were obtained from the Pasteur Institute located in Tehran, Iran, and kept under standard laboratory conditions, which included maintaining a temperature of 23°C ± 2, a 12:12 hours light/dark cycle (with lights turned on at 8:00 a.m.), and ad libitum access to food and water. The day of birth was considered postnatal day zero (PND 0). From PND 2 to PND 14, newborn mice were separated from their mothers for three hours every day (between 10:00 a.m. to 1:00 p.m.). The separated pups were then reintroduced to their mother’s cage from PND 15 until PND 25. Lastly, seventy-five maternally separated male mice were randomly divided into five groups (n = 15). To do this, the pups of each mouse were accidentally numbered on the 25th day after birth (PND = 25) and were randomly assigned to the experimental groups. Additionally, fifteen NMRI male mice that were not subjected to the MS paradigm were selected as the control group. The control group (Group 1) was given normal saline (1 ml/kg). The second to sixth groups were the maternally separated mice treated with normal saline (1 ml/kg), L-arginine (L-arg), a NO precursor, (50 mg/kg), NG-Nitroarginine methyl ester (L-NAME), a NOS inhibitor, (10 mg/kg), ketamine, an NMDA-R antagonist, (0.5 mg/kg), and N-methyl-D-aspartate (NMDA), an NMDA-R agonist, (150 mg/kg), respectively. All drugs were administered intraperitoneally (i.p.) for 1 week from PND 51–53 until PND 58–60. The behavioral experiments were conducted immediately after the completion of the treatments and were performed between 09:00 a.m. and 05:00 p.m. Finally, the mice were sacrificed under deep anesthesia using diethyl ether, and their hippocampi were removed for molecular analysis [28]. Dose and time of administrations were chosen based on previous studies as well as our pilot study [32, 33, 40, 41]. By establishing a bilateral alpha is 0.05 and a confidence interval of 90%, 15 mice were considered for each experimental group based on sample size calculation formula [42]. It has been determined that animals show low subject to subject variation [43]. In order to minimize animal suffering and reduce the number of animals used, based on formula introduced by Charan et al., 5 mice in each group is the acceptable limit and hence can be considered as adequate sample size to see the effect of drug in animal studies [44]. Since, behavioral tests impose different levels of stress to animals, in order to minimize the effect of stress reactivity, from 15 mice in each experimental group, 5 mice subjected to the three chamber sociability test, 5 mice subjected to the shuttle box test and 5 mice subjected to the EPM and MBT. Immediately after behavioral tests, mice were anesthetized with diethyl ether and sacrificed by decapitation and hippocampi samples were harvested for biochemical and molecular evaluations.

### Behavioral tests

#### Three-chamber test

To assess social behaviors, we utilized the three-chamber test. A plexiglass box was partitioned into three chambers, including a middle chamber and two side chambers. Throughout the habituation, sociability, and social preference phases, the mice were given the freedom to explore the box. In the habituation phase, mice were placed in the middle chamber for 5 minutes to acclimatize themselves to the surroundings. To perform the sociability phase, two cylindrical wire cages were put in the two side chambers. During the next stage, one wire cage had a same-sex and same-age mouse that had no prior interaction with the subject mice placed inside it (novel mice 1 or social stimulus 1), and the amount of time spent exploring each chamber was measured for 10 minutes. The other wire cage remained empty. The amount of time spent directly interacting with the social stimulus and the empty chamber (non-social stimulus) was recorded. The sociability index (SI) was calculated as (social stimulus 1—non-social stimulus)/ (social stimulus 1 + non-social stimulus). In the social preference stage (SPI), a novel mouse (new mice 2 or social stimulus 2) with characteristics similar to those of the novel mice 1 was placed in another empty wire cage, and the amount of time spent in each chamber was recorded for 10 minutes. The SPI was determined using the following formula: (social stimulus 2—social stimulus 1)/ (social stimulus 2 + social stimulus 1) [45, 46].

#### Elevated plus-maze (EPM)

To evaluate anxiety-related behaviors in mice, the elevated plus-maze (EPM) was employed. The EPM device was constructed with gray plastic and shaped like a plus sign, with two open arms and two closed arms enclosed by walls facing each other. The platform was raised 50 cm above the ground. Prior to the test, the apparatus was meticulously cleaned with 70% ethanol and allowed to dry. In the test, each mouse was individually placed in the center of the maze and faced one of the closed arms for 5 minutes, allowing it to explore the maze. The time spent in each arm, including the open and closed arms, and the number of entries into each arm were recorded. An arm entry was classified as the placement of all four paws into an arm. Increased time spent in closed arms indicated anxiety-like behavior. After each trial, the maze was cleansed with 70% ethanol to reduce any odor cues left by the mice. The test was conducted in a calm environment with controlled lighting conditions to reduce external disturbances [47].

#### Marble burying test (MBT)

The MBT was performed to evaluate repetitive behavior. To perform the test, each mouse was placed into a clean cage containing 20 marbles (arranged in a uniform grid pattern) and allowed to explore for 20 minutes. The number of marbles buried (covered with at least two-thirds of the bedding) was then counted and recorded for each mouse. The test cage was thoroughly cleaned with 70% ethanol between each test to avoid any potential bias [6, 45].

#### Shuttle box test

To evaluate the passive avoidance memory of mice, the shuttle box apparatus was used. This device includes a bright and a dark chamber, which are connected by a door and have separate grid floors for administering electric shocks. The experiment was conducted over four consecutive days. On the first and second days, the mice were given 5 minutes to explore the apparatus. On the third day, the mice were placed in the bright chamber to begin the acquisition test. After a 2-minute adaptation period, an electric shock (1 mA/second) was delivered to the grid floor of the dark chamber. The latency to enter the dark chamber was measured and recorded. On the fourth day, the mice were placed in the bright chamber again, and the interval between entering the bright chamber and entering the dark chamber (up to 60 seconds) was measured. The apparatus was thoroughly cleaned after each trial to eliminate any potential biases or olfactory cues [6].

#### Biochemical and molecular tests

*Nitrite assay*. The Griess reaction method was utilized to evaluate nitrite levels in the hippocampus. Initially, the mice were anesthetized with diethyl ether and sacrificed, and the hippocampus was removed and immediately placed into liquid nitrogen. Hippocampus homogenates were prepared, and nitrite concentrations were determined using a colorimetric assay based on the Griess reaction. In brief, 100 μL of samples was loaded into each well and mixed with 100 μL of Griess reagent. The automated plate reader measured the absorbance at 540 nm after ten minutes of incubation at room temperature. The level of nitrite was determined using a standard curve of sodium nitrite (Sigma, USA) and normalized to the weight of each sample [48].

*Real-time PCR analysis for the gene expression of nNOS*, *iNOS*, *NR2A*, *and NR2B*. In this study, the gene expression of nNOS, iNOS, NR2A, and NR2B in the hippocampus was assessed using Real-Time PCR. The hippocampus tissue was collected and total RNA was extracted using RNX-plus. The RNA was then reverse-transcribed into cDNA using a PrimeScript RT reagent kit (Takara Bio, Inc., Otsu, Japan). The gene-specific primers and fluorescent probes for nNOS, iNOS, NR2A, and NR2B were designed and optimized. Real-time PCR was performed on the cDNA samples using a light cycler instrument (Roche Diagnostics, Mannheim, Germany) (Takara Bio). The results were analyzed using the 2^-ΔΔCt^ method to calculate the relative gene expression levels of nNOS, iNOS, NR2A, and NR2B in the hippocampus. The housekeeping gene B2M was used as a reference gene to normalize the gene expression levels [28, 49]. Primer sequences present in Table 1.

**Table 1:** The primer sequences used in PCR amplification.

Primers	Forward sequence	Reverse sequence
**B2M**	TCATCGACACCTGAAATCTAGGA	AGGGGTGATACGCTTTACCTTTA
**nNOS**	GGCTGTGCT TTGATGGAG ATGA	AGAATA GGAGGAGAC GCT GT
**iNOS**	CCAACAGGAGAAGGGGACGAA	GGACATCAAAGGTCTCACAGGC
**NR2A**	CTCAGCATTGTCACCTTGGA	GCAGCACTTCTTCACATTCAT
**NR2B**	CTACTGCTGGCTGCTGGTGA	GACTGGAGAATGGAGACGGCTA

### Data analysis

GraphPad PRISM 8 software was used for statistical analysis. Kolmogorov–Smirnov test was applied to evaluate the normal distribution of data. Brown-Forsythe test was used for evaluation of data homogeneity. Analysis showed normal distribution of data with equal variances, thus one-way analysis of variance (ANOVA), a parametric test, followed by Tukey’s post hoc test was used for data analysis. Data presented as Mean± S.E.M. Results were deemed statistically significant at p < 0.05.

## Results

### Behavioral tests

#### Effects of NO/NMDA mediators on sociability and social preference indexes in the three-chamber test

**Fig 1:**
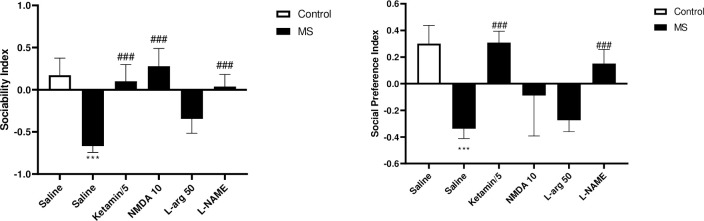
The effect of MS stress and NO/NMDA mediators on sociability index and social preference index. The values were calculated for a sample of 5 mice and reported as the mean ± S.E.M. The statistical analysis employed a one-way ANOVA followed by Tukey’s post-test. ***p<0.001 compared to the control group and ^###^p<0.001 compared to the saline-treated MS group.

Results showed that the MS group exhibited a significant reduction in their SI (p<0.001) in comparison to the control group. However, when MS groups were treated with L-NAME, ketamine, and NMDA, their SI improved significantly (p<0.001). In compared to the saline-treated MS mice (Fig 1). Additionally, we observed that SPI was significantly lower in the MS group in compared to the control group (p<0.001). When MS groups were treated with ketamine and L-NAME their SPI significantly increased compared to the saline-treated MS mice (p<0.001) (Fig 1).

#### Effects of NO/NMDA mediators on repetitive behavior in marbles burying test

**Fig 2:**
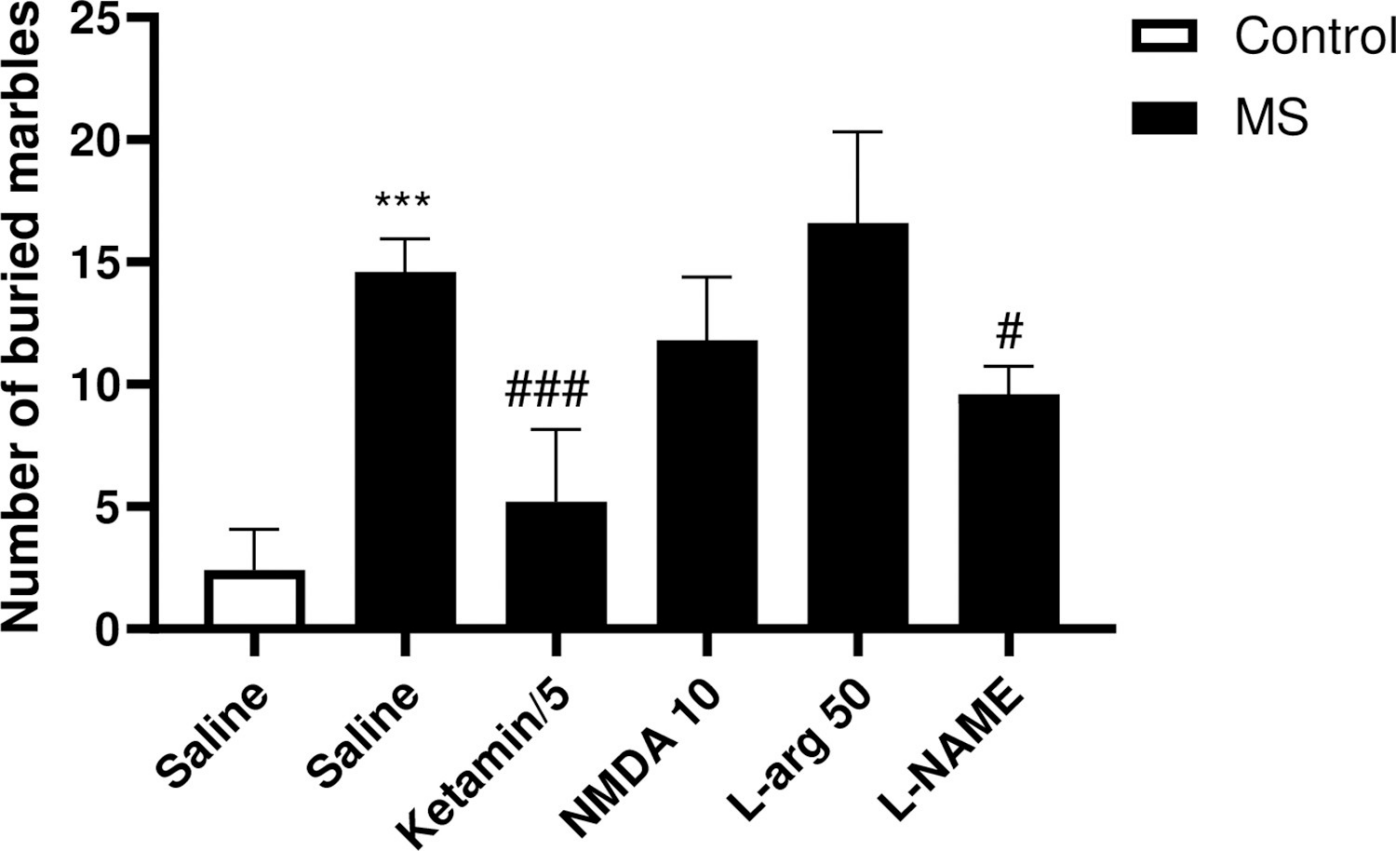
The effect of MS stress and NO/NMDA mediators on the number of marbles buried. The values were calculated for a sample of 5 mice and reported as the mean ± S.E.M. The statistical analysis employed a one-way ANOVA followed by Tukey’s post-test. ***p<0.001 compared to the control group and ^#^p<0.05 and ^###^p<0.001 compared to the saline-treated MS group.

The findings of the study revealed that the MS group had a significantly higher number of buried marbles than the control group (p<0.001). However, the number of marbles buried was significantly decreased in the MS mice treated with L-NAME (p<0.05) and ketamine (p<0.001) compared to those treated with saline (Fig 2).

#### Effects of NO/NMDA mediators on the open arms entries and time in the EPM test

**Fig 3:**
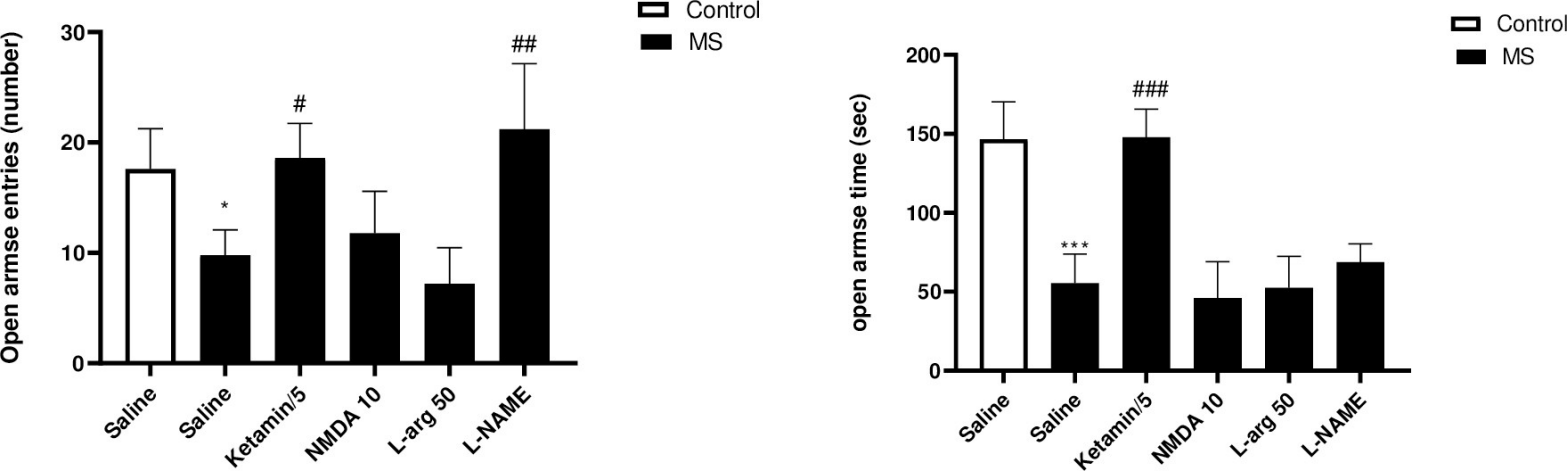
The effect of MS stress and NO/NMDA mediators on the open arms entries and time spent in open arms in the EPM. Values were calculated for a sample of 5 mice and reported as the mean ± S.E.M. The statistical analysis employed a one-way ANOVA followed by Tukey’s post-test. *p<0.05 and ***p<0.001 in comparison to the control group, ^#^p<0.05, ^##^p<0.01 and ^###^p<0.001 in comparison to the saline-treated MS group.

The findings from the EPM test are presented in Fig 3. The MS group demonstrated a notable reduction in the number of open arm entries compared to the control group (p<0.05). Treatment of the MS group with L-NAME (p<0.01) and ketamine (p<0.05) resulted in a significant increase in the number of open arm entries compared to the saline-treated MS mice. Additionally, the time spent in open arms was significantly lower in the MS group when compared to the control group (p<0.001). However, administering ketamine to the MS group resulted in a significant increase in the time spent in open arms compared to the saline-treated MS mice (p<0.001).

#### Effects of NO/NMDA mediators on the passive avoidance memory in the shuttle box test

**Fig 4:**
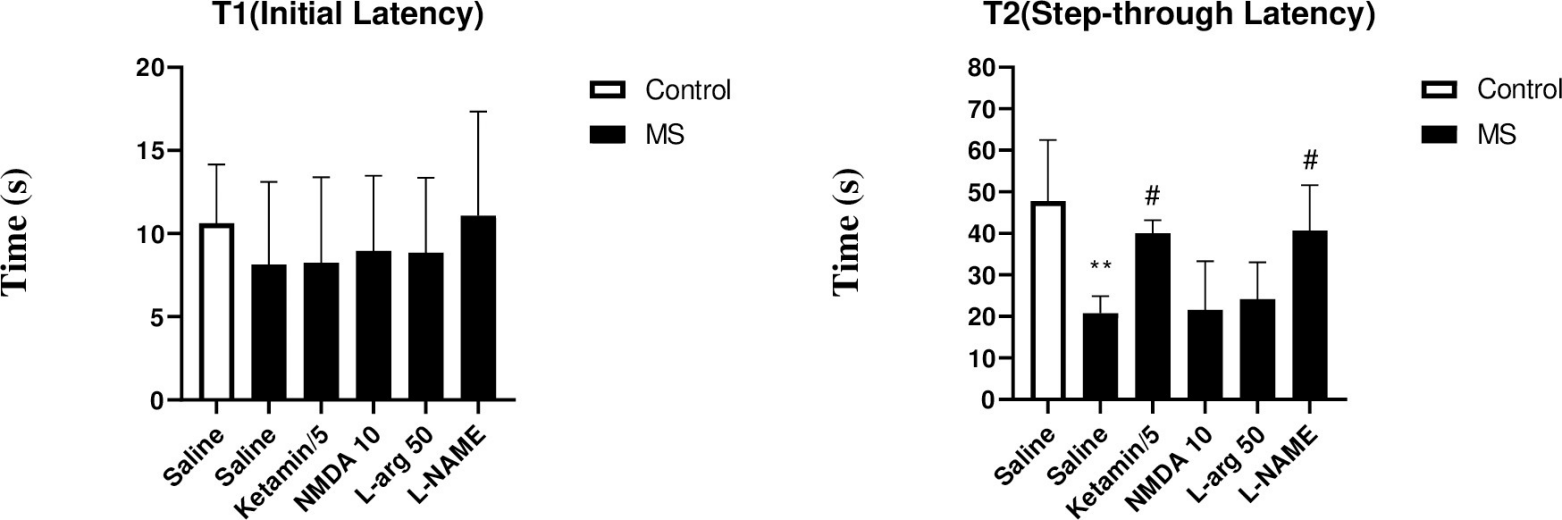
The effect of MS stress and NO/NMDA mediators on the initial latency and step-through latency in the passive avoidance response in the shuttle box test. Values were calculated for a sample of 5 mice and reported as the mean ± S.E.M. The statistical analysis employed a one-way ANOVA followed by Tukey’s post-test. **p<0.001 in compared to the control group, ^#^p<0.05 in compared to the saline-treated MS group.

The results showed that there was no notable difference in the initial phase (T1) of the shuttle box test between experimental groups. However, in the subsequent phase of the test (T2) (step-through latency), results showed markedly reduced in the MS group compared to the control group (p<0.01). Interestingly, the administration of ketamine (p<0.05), and L-NAME (p<0.05) significantly increased the T2 compared to the saline-treated MS group (Fig 4).

### Biochemical/Molecular analyses

#### Effects of NO/NMDA mediators on the nitrite levels in the hippocampus

**Fig 5:**
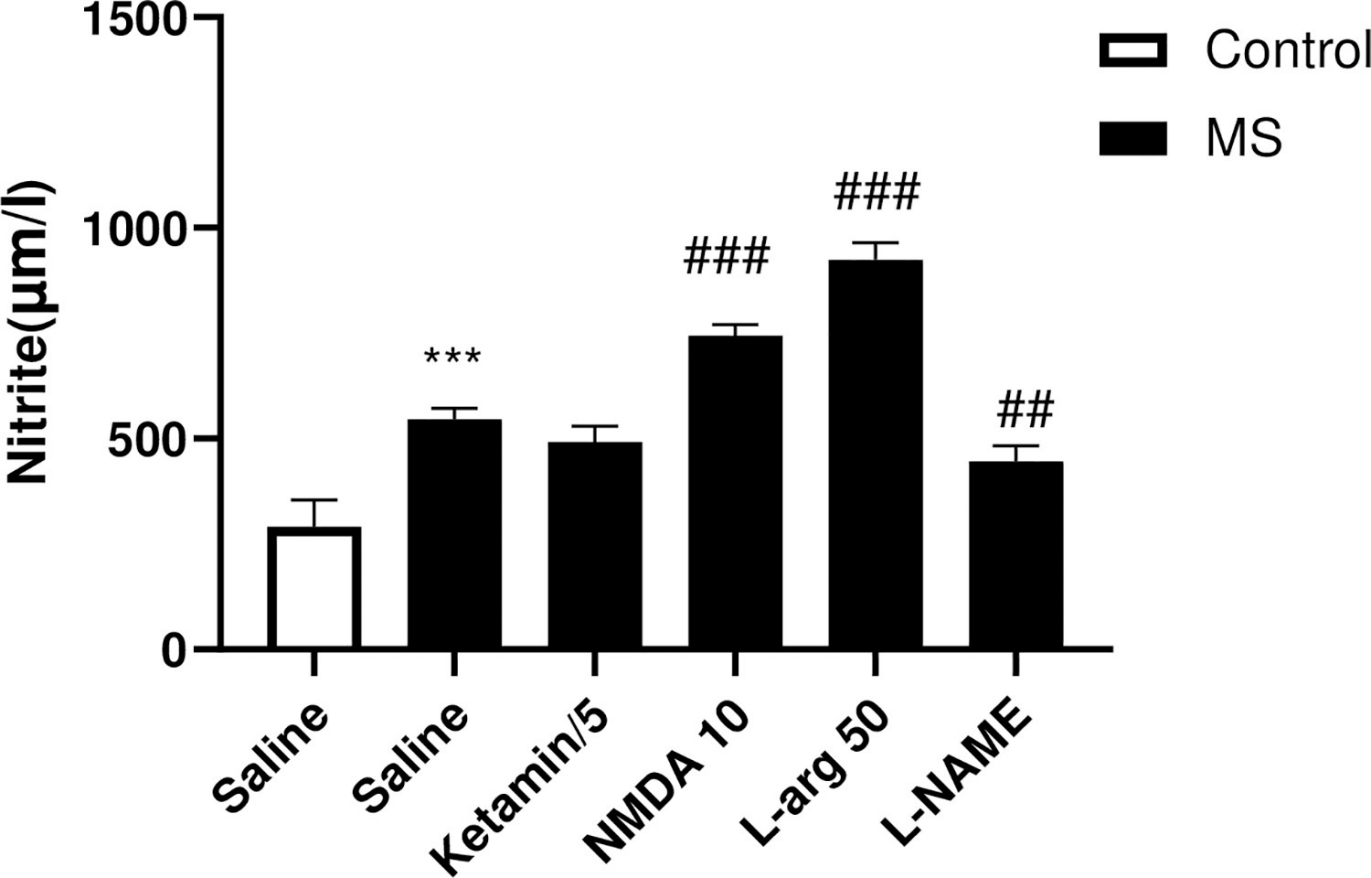
The effect of MS stress and NO/NMDA mediators on the nitrite levels in the hippocampus. Values were calculated for a sample of 5 mice and reported as the mean ± S.E.M. The statistical analysis employed a one-way ANOVA followed by Tukey’s post-test. ***p<0.001 compared to the control group, ^##^p<0.01 and ^###^p<0.001 in compared to the saline-treated MS group.

The results indicated a significant increase in nitrite levels in the hippocampus tissue of the MS group compared to the control group (p<0.001). Treatment of MS mice with L-arg (p<0.001) and NMDA (p<0.001) resulted in a significant elevation of nitrite levels compared to the saline-treated MS mice. Conversely, the administration of L-NAME to the MS group significantly decreased the hippocampal nitrite levels compared to the saline-treated MS mice (p<0.01) (Fig 5).

#### The gene expression of iNOS, nNOS, NR2A, and NR2B in the hippocampus following administration of NO/NMDA mediators

**Fig 6:**
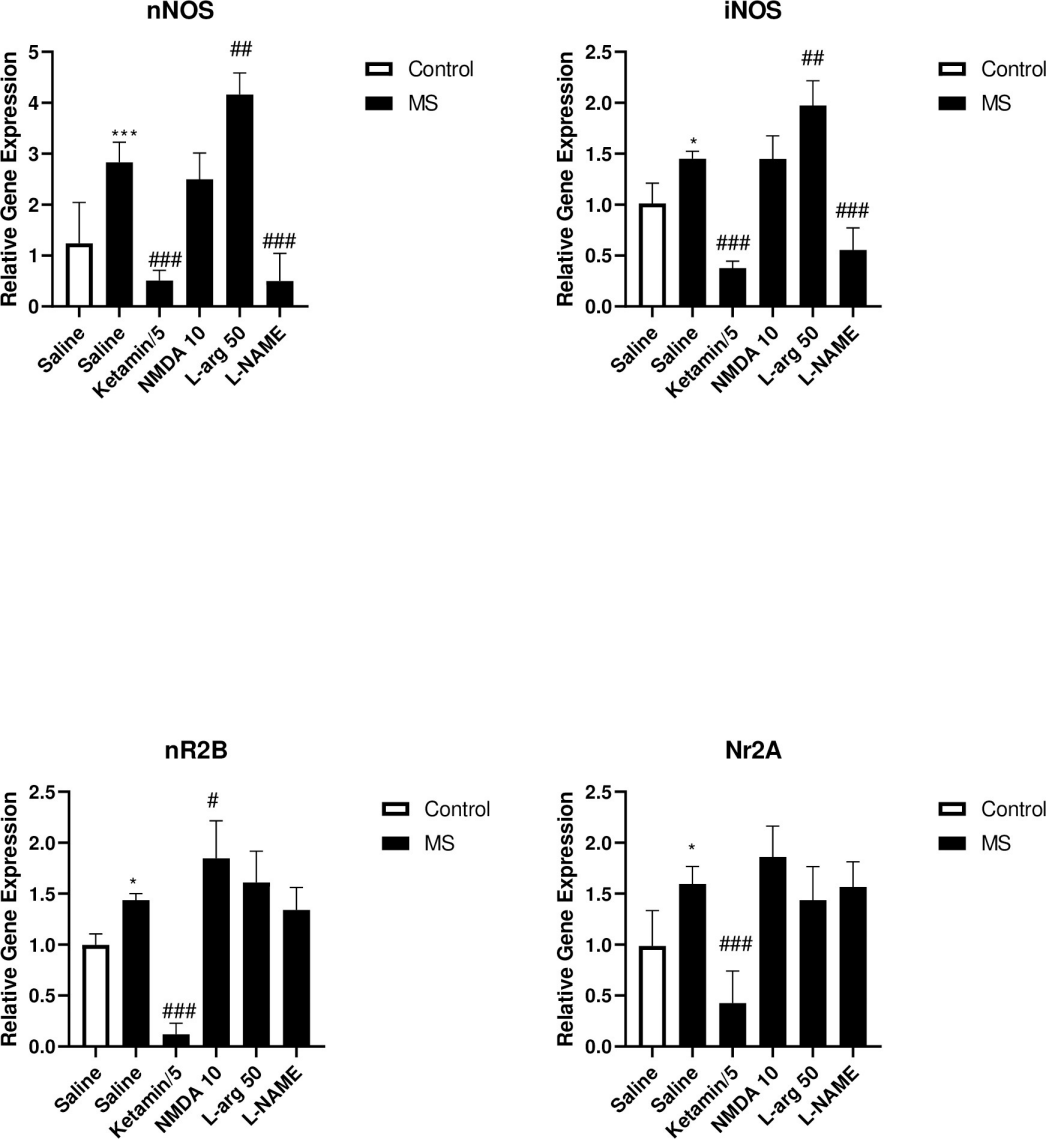
The effect of MS stress and NO/NMDA mediators on the gene expression of iNOS, nNOS, NR2A, and NR2B in the hippocampus. Values were calculated for a sample of 5 mice and reported as the mean ± S.E.M. The statistical analysis employed one-way ANOVA followed by Tukey’s post-test. *p<0.05 and ***p<0.001 in comparison to the control group, ^#^p<0.05, ^##^p<0.01 and ^###^p<0.001 in comparison to the saline-treated MS group.

Fig 6 shows the effects of NO/NMDA mediators on the gene expression of iNOS, nNOS, NR2A, and NR2B in the hippocampus.

The gene expression of iNOS significantly increased in the MS group compared to the control group (p<0.01). Treatment of the MS group with L-NAME (p<0.001) and ketamine (p<0.001) significantly decreased, while treatment with L-arg (p<0.01) significantly increased the gene expression of iNOS compared to the saline-treated MS mice.

The gene expression of nNOS significantly increased in the MS group compared to the control group (p<0.001). Treatment of the MS mice with L-NAME (p<0.001) and ketamine (p<0.001) significantly decreased, while administration of L-arg (p<0.01) significantly increased the gene expression of nNOS compared to the saline-treated MS mice.

We found that the gene expression of NR2A significantly increased in the MS group compared to the control group (p<0.05). Treatment of the MS group with ketamine significantly decreased the gene expression of NR2A compared to the saline-treated MS mice (p<0.001).

Results showed that the gene expression of NR2B significantly increased in the MS group compared to the control group (p<0.05). Treatment of the MS group with ketamine significantly decreased the gene expression of NR2B compared to the saline-treated MS mice (p<0.001). Furthermore, treatment of MS animals with NMDA significantly increased the gene expression of NR2B compared to the saline-treated MS mice (p<0.05).

## Discussion

The aim of this study was to investigate the potential role of the NO/NMDA pathway in the development of autistic-like behaviors induced by MS in mice. The significant findings of this study revealed that MS is associated with the development of autistic-like behaviors in adult male mice. Specifically, we observed a decrease in SI and SPI in the three-chamber sociability test, an increased number of buried marbles in the MBT, decreased step-through latency (T2) in the shuttle box test, as well as reduced open arm time and open arm entries in the EPM. These behavioral changes were accompanied by an increase in nitrite levels and the expression of iNOS, nNOS, NR2A, and NR2B genes in the hippocampus. Importantly, treatment with NOS/NMDA-R inhibitors (L-NAME and ketamine) attenuated the negative effects of MS on autistic-like behaviors, decreased nitrite levels, and suppressed the expression of iNOS, nNOS, NR2A, and NR2B genes in the hippocampus. Conversely, administration of NOS/NMDA-R activators (L-arg and NMDA) worsened the negative effects of MS on behavior and increased nitrite levels and the expression of iNOS, nNOS, NR2A, and NR2B genes in the hippocampus.

An early direct relationship between infant and mother plays a main role in developing the nervous system [50, 51]. Many previous studies have reported that early life adversities have enduring and long-lasting negative effects on behavioral, psychological, and neurological features in adulthood [29, 52, 53]. It has been demonstrated that maternal separation (MS) stress as a valid adverse experience in early life significantly increased the risk of neurological and psychiatric disorders in adulthood [54, 55]. MS, with its structural, developmental, neuroendocrine, and neurochemical changes in different areas of the central nervous system, such as the hippocampus causes mood-behavioral disorders. These changes provide the basis for the incidence of neurodegenerative diseases in adulthood [56]. Studies on ASD designated defects in brain development as well as structural disturbances [57]. Previous evidence showed that experiencing early life difficulties like MS stress could lead to the development of ASD [6, 58]. MS through changes in neuronal plasticity triggers the development of ASD [59]. It has been shown that modulating neurochemical and neurostructural changes in maternally separated animals could ameliorate autistic-like behaviors [60]. Moreover, the presence of heightened oxidative stress and a lack of sufficient antioxidant protection can exacerbate autistic-like behaviors. However, it is possible to alleviate these behaviors by boosting neuronal antioxidants [61]. Besides, the pivotal role of neuroinflammation in the pathogenesis of ASD has been extensively documented, revealing intriguing insights into the complex etiology of the disorder. A relevant study conducted by Sheikh et al. provides compelling evidence regarding the potential efficacy of Peroxisome proliferator-activated receptor delta (PPARδ) activation in alleviating behavioral dysfunction in an autistic mouse model. Their research demonstrated that PPARδ, known for its anti-inflammatory effects in animal models of neuroinflammatory diseases, exhibited promising therapeutic effects. By activating PPARδ, which is associated with anti-inflammatory properties, Sheikh et al. observed significant improvements in behavioral dysfunction in the mouse model of autism [62]. In this regard, in the orbitofrontal cortex of autistic patients, post-mortem analyses have unveiled dysregulated expression of NF-κB, a key transcription factor associated with inflammatory processes. Notably, heightened activity of microglia, the resident immune cells of the central nervous system, has been observed in conjunction with this aberrant NF-κB expression [63]. Compellingly, proinflammatory cytokines, including tumor necrosis factor-alpha (TNF-α), interleukin-6 (IL-6), IFN-γ, have exhibited significant upregulation in the brains of individuals with autism [64]. Concurrently, investigations have revealed that MS stress can elicit neuroinflammatory responses within the brain [65]. Consequently, this induced neuroinflammation may serve as one of pivotal triggers for the emergence and progression of autistic-like behaviors in a mouse model of MS stress.

The main symptoms of ASD are defects in social interaction and communication, as well as repetitive and stereotyped behaviors [66, 67]. In addition, it has been well-determined that autism is associated with anxiety and memory disturbance [68, 69]. Preclinical studies have determined that ASD is associated with impaired passive avoidance memory in the shuttle box [6, 70]. In addition, evidence showed that ASD is associated with a decrease in the time and number of entries in the open arms of the EMP, indicating that ASD is accompanied by anxiety-like behaviors [6, 45, 71]. In the case of repetitive behaviors, previous studies have shown that autistic mice buried more marbles than healthy mice, indicating obsessive-compulsive and repetitive behaviors in autistic mice [72, 73]. Furthermore, preclinical studies have demonstrated that ASD is linked with deficits in social abilities and social interactions in the three-chamber sociability test [74]. The findings of the present study showed that experiencing early life stress as MS is associated with autistic-like behaviors in the adult male mice. We found that MS mice have lower SI and SPI in the three-chamber sociability test and buries more marbles in the MBT than control mice. In addition, we observed that MS stress decreased step-through latency (T2) in the shuttle box as well as decreased open arms time and entries in the EPM. Our results are in line with aforementioned studies and indicating that MS stress caused autistic-like behaviors.

Prior studies have established that NO functions as a dual-acting agent in the CNS [75]. While excessive amounts of NO can lead to neuronal damage and cell death [76], maintaining physiological levels of NO is crucial for regulating neurological functions [77]. The precise role of NO in autism is an active area of research that remains unclear. However, dysregulated NO levels in the brain may contribute to the development of neurological disorders, including autism [35]. Elevated NO levels can interact with oxygen to produce reactive nitrogen species (RNS), leading to oxidative stress and lipid peroxidation [78]. Moreover, NO plays a critical role in cellular signal transduction through the SNO mechanism [79]. Recent studies have suggested that NO and SNO-modified proteins are involved in the pathophysiology of various neurological disorders, including autism spectrum disorder (ASD). A network of SNO-modified proteins may play a functional role in the synaptic vesicle cycle, neurotransmission, and glutamatergic pathway, all of which are implicated in the development of ASD [80]. Results of the present study showed that MS stress is associated with an increase in nitrite levels in the hippocampus. Furthermore, we demonstrated a rise in the gene expression of iNOS and nNOS in the hippocampus of MS mice. Administration of the NOS inhibitor, L-NAME, to the MS mice significantly decreased levels of nitrite and also decrease the gene expression of iNOS and nNOS in the hippocampus. While administration of NO precursor, L-arg, to the MS mice increased levels of nitrite and the gene expression of iNOS and nNOS. In the case of behavioral assessments, we observed that administration of L-NAME increased SI and SPI in the three-chamber sociability test and decreased the number of buried marbles in the MBT as well as increased step-through latency (T2), in the shuttle box and increased open arms time and entries in the EPM. While treatment of MS mice with L-arg increased levels of nitrite as well as gene expression of iNOS and nNOS in the hippocampus and also worsen the negative effects of MS on autistic-like behaviors. Considering the bilateral role of NO and NMDA in regulating each other [32], we found that the administration of ketamine, an NMDA-R antagonist, decreased the expression of iNOS and nNOS genes. In this concept, Alqahtani and colleagues demonstrated that ketamine is able to reduce iNOS gene expression in mice with traumatic brain injury [81]. Our finding is in line with the abovementioned studies that determined the role of NO dysregulation in ASD. These results, partially at least, indicated that the NO pathway may mediate the development of autistic-like behaviors in the MS stress model in mice.

Previous research has shown that the function of NMDA-R plays a critical role in the regulation of social behavior [82]. In both human and animal models, dysfunction of NMDA-R at excitatory synapses has been associated with significant autistic-like manifestations [18]. It has been determined that NMDA-R agonists could ameliorate autistic symptoms [19]. In this regard, Won et al., have shown that enhancing NMDA-R function can improve autistic-like behavior in genetically mutated mice (Shank2-mutant) [83]. On the contrary, some evidence demonstrated that elevated NMDAR function is also involved in ASDs and administration of NMDA-R antagonists improved ASD-related symptoms [20, 21]. Several previous studies have shown that MS increased the gene expression of NMDA-R subunits (NR2A and NR2B) in the brain [84, 85]. In agreement with the aforementioned previous studies, in this study, we found that MS is associated with an increase in the gene expression of NR2A and NR2B in the hippocampus. Administration of the NMDA-R antagonist, ketamine, to the MS mice significantly decrease the gene expression of NR2A and NR2B in the hippocampus. While administration of NMDA-R agonist, NMDA, to the MS mice increased levels of nitrite and the gene expression of NR2B in the hippocampus. In the case of behavioral tests, we observed that administration of ketamine increased SI and SPI in the three-chamber sociability test and decreased the number of buried marbles in the MBT as well as increased step-through latency (T2) in the shuttle box, and increased open arms time and entries in the EPM.

Results of this study showed that autistic-like behaviors induced by the MS stress paradigm are due to, partially at least, dysregulation of the NO/NMDA pathway in the hippocampus.

One of the limitations of this study is that the molecular mechanisms underlying the effects of NO/NMDA mediators evaluated on the gene expression level and it therefore requires further investigations to evaluate these effects on the protein levels (using western-blotting, IHC or ELISA). Another limitation of this study is that we did not examine the effects of NO/NMDA mediators in autistic-like behaviors following MS in female mice.

## Conclusions

In conclusion, the findings of this study provide evidence supporting the involvement of the NO/NMDA pathway in the development of autistic-like behaviors triggered by MS stress in adult male mice. Our results suggest that the dysregulation of this pathway in the hippocampus contributes, at least partially, to the manifestation of autistic-like behaviors observed in this model.

## Supporting information

S1 Data Raw data of experiments presents in excel form. (XLSX)

## References

[1] Berry K, Russell K, Frost K. Restricted and Repetitive Behaviors in Autism Spectrum Disorder: a Review of Associated Features and Presentation Across Clinical Populations. Current Developmental Disorders Reports. 2018;5(2):108–15. doi: 10.1007/s40474-018-0139-0

[2] Bölte S, Girdler S, Marschik PB. The contribution of environmental exposure to the etiology of autism spectrum disorder. Cellular and Molecular Life Sciences. 2019;76(7):1275–97. doi: 10.1007/s00018-018-2988-4 30570672 PMC6420889

[3] Own LS, Patel PD. Maternal behavior and offspring resiliency to maternal separation in c57bl/6 mice. Hormones and Behavior. 2013;63(3):411–7. doi: 10.1016/j.yhbeh.2012.11.010 23195752

[4] Haratizadeh S, Parvan M, Mohammadi S, Shabani M, Nozari M. An overview of modeling and behavioral assessment of autism in the rodent. International Journal of Developmental Neuroscience. 2021;81(3):221–8. doi: 10.1002/jdn.10096 33570815

[5] Makris G, Eleftheriades A, Pervanidou P. Early Life Stress, Hormones, and Neurodevelopmental Disorders. Hormone Research in Paediatrics. 2023;96(1):17–24. doi: 10.1159/000523942 35259742

[6] Farzan M, Farzan M, Amini-Khoei H, Shahrani M, Bijad E, Anjomshoa M, et al. Protective effects of vanillic acid on autistic-like behaviors in a rat model of maternal separation stress: Behavioral, electrophysiological, molecular and histopathological alterations. International Immunopharmacology. 2023;118:110112. doi: 10.1016/j.intimp.2023.110112 37030116

[7] Bourgeron T. From the genetic architecture to synaptic plasticity in autism spectrum disorder. Nature Reviews Neuroscience. 2015;16(9):551–63. doi: 10.1038/nrn3992 26289574

[8] Reshetnikov VV, Lepeshko AA, Ryabushkina YA, Studenikina AA, Merkulova TI, Bondar NP. The Long-Term Effects of Early Postnatal Stress on Cognitive Abilities and Expression of Genes of the Glutamatergic System in Mice. Neurochemical Journal. 2018;12(2):142–51. doi: 10.1134/S1819712418020095

[9] Choudhury PR, Lahiri S, Rajamma U. Glutamate mediated signaling in the pathophysiology of autism spectrum disorders. Pharmacology Biochemistry and Behavior. 2012;100(4):841–9. doi: 10.1016/j.pbb.2011.06.023 21756930

[10] Warburton EC, Barker GRI, Brown MW. Investigations into the involvement of NMDA mechanisms in recognition memory. Neuropharmacology. 2013;74:41–7. doi: 10.1016/j.neuropharm.2013.04.013 23665343 PMC3895175

[11] Willard SS, Koochekpour S. Glutamate, glutamate receptors, and downstream signaling pathways. Int J Biol Sci. 2013;9(9):948–59. Epub 20130922. doi: 10.7150/ijbs.6426 ; PubMed Central PMCID: PMC3805900. 24155668 PMC3805900

[12] Paoletti P, Bellone C, Zhou Q. NMDA receptor subunit diversity: impact on receptor properties, synaptic plasticity and disease. Nature Reviews Neuroscience. 2013;14(6):383–400. doi: 10.1038/nrn3504 23686171

[13] Amiri S, Alijanpour S, Tirgar F, Haj-Mirzaian A, Amini-Khoei H, Rahimi-Balaei M, et al. NMDA receptors are involved in the antidepressant-like effects of capsaicin following amphetamine withdrawal in male mice. Neuroscience. 2016;329:122–33. doi: 10.1016/j.neuroscience.2016.05.003 27167081

[14] Yashiro K, Philpot BD. Regulation of NMDA receptor subunit expression and its implications for LTD, LTP, and metaplasticity. Neuropharmacology. 2008;55(7):1081–94. doi: 10.1016/j.neuropharm.2008.07.046 18755202 PMC2590778

[15] Nisar S, Bhat AA, Masoodi T, Hashem S, Akhtar S, Ali TA, et al. Genetics of glutamate and its receptors in autism spectrum disorder. Molecular Psychiatry. 2022;27(5):2380–92. doi: 10.1038/s41380-022-01506-w 35296811 PMC9135628

[16] Burket JA, Herndon AL, Winebarger EE, Jacome LF, Deutsch SI. Complex effects of mGluR5 antagonism on sociability and stereotypic behaviors in mice: Possible implications for the pharmacotherapy of autism spectrum disorders. Brain Research Bulletin. 2011;86(3):152–8. doi: 10.1016/j.brainresbull.2011.08.001 21840381

[17] Gandal MJ, Anderson RL, Billingslea EN, Carlson GC, Roberts TPL, Siegel SJ. Mice with reduced NMDA receptor expression: more consistent with autism than schizophrenia? Genes, Brain and Behavior. 2012;11(6):740–50. doi: 10.1111/j.1601-183X.2012.00816.x 22726567 PMC3808979

[18] Lee E-J, Choi SY, Kim E. NMDA receptor dysfunction in autism spectrum disorders. Current Opinion in Pharmacology. 2015;20:8–13. doi: 10.1016/j.coph.2014.10.007 25636159

[19] Urbano M, Okwara L, Manser P, Hartmann K, Herndon A, Deutsch SI. A trial of D-cycloserine to treat stereotypies in older adolescents and young adults with autism spectrum disorder. Clinical neuropharmacology. 2014;37(3):69. doi: 10.1097/WNF.0000000000000033 24824660 PMC4354861

[20] Hosenbocus S, Chahal R. Memantine: a review of possible uses in child and adolescent psychiatry. Journal of the Canadian Academy of Child and Adolescent Psychiatry. 2013;22(2):166. 23667364 PMC3647634

[21] Elnaiem W, Benmelouka AY, Elgendy AMN, Abdelgalil MS, Brimo Alsaman MZ, Mogheeth A, et al. Evaluation of memantine’s efficacy and safety in the treatment of children with autism spectrum disorder: A systematic review and meta‐analysis. Human Psychopharmacology: Clinical and Experimental. 2022;37(5):e2841. 10.1002/hup.2841 35315131

[22] Bielau H, Brisch R, Bernard-Mittelstaedt J, Dobrowolny H, Gos T, Baumann B, et al. Immunohistochemical evidence for impaired nitric oxide signaling of the locus coeruleus in bipolar disorder. Brain Research. 2012;1459:91–9. doi: 10.1016/j.brainres.2012.04.022 22560594

[23] Xu W, Liu LZ, Loizidou M, Ahmed M, Charles IG. The role of nitric oxide in cancer. Cell Research. 2002;12(5):311–20. doi: 10.1038/sj.cr.7290133 12528889

[24] Hassanipour M, Amini-Khoei H, Shafaroodi H, Shirzadian A, Rahimi N, Imran-Khan M, et al. Atorvastatin attenuates the antinociceptive tolerance of morphine via nitric oxide dependent pathway in male mice. Brain research bulletin. 2016;125:173–80. doi: 10.1016/j.brainresbull.2016.07.002 27381980

[25] Król M, Kepinska M. Human Nitric Oxide Synthase—Its Functions, Polymorphisms, and Inhibitors in the Context of Inflammation, Diabetes and Cardiovascular Diseases. International Journal of Molecular Sciences. 2021;22(1):56. doi: 10.3390/ijms22010056 33374571 PMC7793075

[26] Hollas MA, Ben Aissa M, Lee SH, Gordon-Blake JM, Thatcher GRJ. Pharmacological manipulation of cGMP and NO/cGMP in CNS drug discovery. Nitric Oxide. 2019;82:59–74. Epub 20181028. doi: 10.1016/j.niox.2018.10.006 ; PubMed Central PMCID: PMC7645969. 30394348 PMC7645969

[27] Hamoudi W, Tripathi MK, Ojha SK, Amal H. A cross-talk between nitric oxide and the glutamatergic system in a Shank3 mouse model of autism. Free Radical Biology and Medicine. 2022;188:83–91. doi: 10.1016/j.freeradbiomed.2022.06.007 35716826

[28] Lorigooini Z, Boroujeni SN, Sayyadi-Shahraki M, Rahimi-Madiseh M, Bijad E, Amini-Khoei H. Limonene through attenuation of neuroinflammation and nitrite level exerts antidepressant-like effect on mouse model of maternal separation stress. Behavioural neurology. 2021;2021. doi: 10.1155/2021/8817309 33564342 PMC7864762

[29] Lorigooini Z, Dehsahraei KS, Bijad E, Dehkordi SH, Amini-Khoei H. Trigonelline through the attenuation of oxidative stress exerts antidepressant-and anxiolytic-like effects in a mouse model of maternal separation stress. Pharmacology. 2020;105(5–6):289–99. doi: 10.1159/000503728 31630147

[30] Amini-Khoei H, Nasiri Boroujeni S, Maghsoudi F, Rahimi-Madiseh M, Bijad E, Moradi M, et al. Possible involvement of l-arginine-nitric oxide pathway in the antidepressant activity of Auraptene in mice. Behavioral and Brain Functions. 2022;18(1):1–9. 35164803 10.1186/s12993-022-00189-1 PMC8842875

[31] Haj-Mirzaian A, Amiri S, Amini-Khoei H, Haj-Mirzaian A, Hashemiaghdam A, Ramezanzadeh K, et al. Involvement of NO/NMDA-R pathway in the behavioral despair induced by amphetamine withdrawal. Brain research bulletin. 2018;139:81–90. doi: 10.1016/j.brainresbull.2018.02.001 29421244

[32] Lorigooini Z, Salimi N, Soltani A, Amini-Khoei H. Implication of NMDA-NO pathway in the antidepressant-like effect of ellagic acid in male mice. Neuropeptides. 2019;76:101928. doi: 10.1016/j.npep.2019.04.003 31078318

[33] Haj-Mirzaian A, Ramezanzadeh K, Tafazolimoghadam A, Kazemi K, Nikbakhsh R, Nikbakhsh R, et al. Protective effect of minocycline on LPS-induced mitochondrial dysfunction and decreased seizure threshold through nitric oxide pathway. European journal of pharmacology. 2019;858:172446. doi: 10.1016/j.ejphar.2019.172446 31202800

[34] Mehta R, Kuhad A, Bhandari R. Nitric oxide pathway as a plausible therapeutic target in autism spectrum disorders. Expert Opinion on Therapeutic Targets. 2022;26(7):659–79. doi: 10.1080/14728222.2022.2100252 35811505

[35] Yui K, Kawasaki Y, Yamada H, Ogawa S. Oxidative stress and nitric oxide in autism spectrum disorder and other neuropsychiatric disorders. CNS & Neurological Disorders-Drug Targets (Formerly Current Drug Targets-CNS & Neurological Disorders). 2016;15(5):587–96. doi: 10.2174/1871527315666160413121751 27071787

[36] Söğüt S, Zoroğlu SS, Özyurt H, Yılmaz HR, Özuğurlu F, Sivaslı E, et al. Changes in nitric oxide levels and antioxidant enzyme activities may have a role in the pathophysiological mechanisms involved in autism. Clinica Chimica Acta. 2003;331(1–2):111–7. doi: 10.1016/s0009-8981(03)00119-0 12691871

[37] Ahmad SF, Ansari MA, Nadeem A, Alzahrani MZ, Bakheet SA, Attia SM. Resveratrol Improves Neuroimmune Dysregulation Through the Inhibition of Neuronal Toll-Like Receptors and COX-2 Signaling in BTBR T+ Itpr3tf/J Mice. NeuroMolecular Medicine. 2018;20(1):133–46. doi: 10.1007/s12017-018-8483-0 29468499

[38] Ahmad SF, Ansari MA, Nadeem A, Bakheet SA, Alshammari MA, Khan MR, et al. S3I-201, a selective Stat3 inhibitor, restores neuroimmune function through upregulation of Treg signaling in autistic BTBR T+ Itpr3tf/J mice. Cellular Signalling. 2018;52:127–36. doi: 10.1016/j.cellsig.2018.09.006 30213685

[39] Ahmad SF, Ansari MA, Nadeem A, Bakheet SA, Al-Ayadhi LY, Attia SM. Toll-like receptors, NF-κB, and IL-27 mediate adenosine A2A receptor signaling in BTBR T+ Itpr3tf/J mice. Progress in Neuro-Psychopharmacology and Biological Psychiatry. 2017;79:184–91. doi: 10.1016/j.pnpbp.2017.06.034 28668513

[40] Ghasemi M, Shafaroodi H, Nazarbeiki S, Meskar H, Ghasemi A, Bahremand A, et al. Inhibition of NMDA receptor/NO signaling blocked tolerance to the anticonvulsant effect of morphine on pentylenetetrazole-induced seizures in mice. Epilepsy research. 2010;91(1):39–48. doi: 10.1016/j.eplepsyres.2010.06.010 20663644

[41] Ostadhadi S, Ahangari M, Nikoui V, Norouzi-Javidan A, Zolfaghari S, Jazaeri F, et al. Pharmacological evidence for the involvement of the NMDA receptor and nitric oxide pathway in the antidepressant-like effect of lamotrigine in the mouse forced swimming test. Biomedicine & Pharmacotherapy. 2016;82:713–21. doi: 10.1016/j.biopha.2016.05.035 27470415

[42] Chen Y, Cai W, Li C, Su Z, Guo Z, Li Z, et al. Sex differences in peripheral monoamine transmitter and related hormone levels in chronic stress mice with a depression-like phenotype. PeerJ. 2022;10:e14014. doi: 10.7717/peerj.14014 36132219 PMC9484450

[43] Jiang Z-D, Alexander A, Ke S, Valilis EM, Hu S, Li B, et al. Stability and efficacy of frozen and lyophilized fecal microbiota transplant (FMT) product in a mouse model of Clostridium difficile infection (CDI). Anaerobe. 2017;48:110–4. doi: 10.1016/j.anaerobe.2017.08.003 28801119

[44] Charan J, Kantharia N. How to calculate sample size in animal studies? Journal of Pharmacology and Pharmacotherapeutics. 2013;4(4):303–6. doi: 10.4103/0976-500X.119726 24250214 PMC3826013

[45] Amini F, Amini-Khoei H, Haratizadeh S, Setayesh M, Basiri M, Raeiszadeh M, et al. Hydroalcoholic extract of Passiflora incarnata improves the autistic-like behavior and neuronal damage in a valproic acid-induced rat model of autism. Journal of Traditional and Complementary Medicine. 2023. 10.1016/j.jtcme.2023.02.005 PMC10310915 37396155

[46] Pang R, Yan S, Tu Y, Qian S, Yu H, Hu X, et al. Transient hearing abnormalities precede social deficits in a mouse model of autism. Behavioural Brain Research. 2023;437:114149. doi: 10.1016/j.bbr.2022.114149 36206820

[47] Sadeghi MA, Hemmati S, Yousefi-Manesh H, Fekrvand S, Foroutani L, Nassireslami E, et al. Neuronal nitric oxide synthase inhibition accelerated the removal of fluoxetine’s anxiogenic activity in an animal model of PTSD. Behavioural Brain Research. 2023;437:114128. doi: 10.1016/j.bbr.2022.114128 36174841

[48] Amiri S, Haj-Mirzaian A, Rahimi-Balaei M, Razmi A, Kordjazy N, Shirzadian A, et al. Co-occurrence of anxiety and depressive-like behaviors following adolescent social isolation in male mice; possible role of nitrergic system. Physiology & behavior. 2015;145:38–44. doi: 10.1016/j.physbeh.2015.03.032 25817356

[49] Omidi-Ardali H, Lorigooini Z, Soltani A, Balali-Dehkordi S, Amini-Khoei H. Inflammatory responses bridge comorbid cardiac disorder in experimental model of IBD induced by DSS: protective effect of the trigonelline. Inflammopharmacology. 2019;27:1265–73. doi: 10.1007/s10787-019-00581-w 30924005

[50] Johnson M, Deardorff J, Davis EL, Martinez W, Eskenazi B, Alkon A. The relationship between maternal responsivity, socioeconomic status, and resting autonomic nervous system functioning in Mexican American children. International Journal of Psychophysiology. 2017;116:45–52. doi: 10.1016/j.ijpsycho.2017.02.010 28238817 PMC5446802

[51] Lapp HE, Champagne FA. Rodent Models for Studying the Impact of Variation in Early Life Mother–Infant Interactions on Mood and Anxiety. Psychiatric Vulnerability, Mood, and Anxiety Disorders: Tests and Models in Mice and Rats: Springer; 2022. p. 309–28.

[52] de Souza JA, da Silva MC, Junior JCdSF, de Souza FL, de Souza SL. Maternal separation in the light or dark phase of the circadian cycle has different effects on the corticosterone levels and anxiety-like behavior in male adult rats. Physiology & Behavior. 2022;247:113725. 35108569 10.1016/j.physbeh.2022.113725

[53] Calpe-López C, Martínez-Caballero M, García-Pardo M, Aguilar M. Brief maternal separation inoculates against the effects of social stress on depression-like behavior and cocaine reward in mice. Frontiers in Pharmacology. 2022;13:224. doi: 10.3389/fphar.2022.825522 35359840 PMC8961977

[54] Huang J, Shen C, Ye R, Shi Y, Li W. The effect of early maternal separation combined with adolescent chronic unpredictable mild stress on behavior and synaptic plasticity in adult female rats. Frontiers in Psychiatry. 2021;12:539299. doi: 10.3389/fpsyt.2021.539299 33746787 PMC7973020

[55] Nouri A, Hashemzadeh F, Soltani A, Saghaei E, Amini-Khoei H. Progesterone exerts antidepressant-like effect in a mouse model of maternal separation stress through mitigation of neuroinflammatory response and oxidative stress. Pharmaceutical Biology. 2020;58(1):64–71. doi: 10.1080/13880209.2019.1702704 31873049 PMC6968520

[56] Tanaka T, Hirai S, Hosokawa M, Saito T, Sakuma H, Saido T, et al. Early-life stress induces the development of Alzheimer’s disease pathology via angiopathy. Experimental neurology. 2021;337:113552. doi: 10.1016/j.expneurol.2020.113552 33309748

[57] Van Rooij D, Anagnostou E, Arango C, Auzias G, Behrmann M, Busatto GF, et al. Cortical and subcortical brain morphometry differences between patients with autism spectrum disorder and healthy individuals across the lifespan: results from the ENIGMA ASD Working Group. American Journal of Psychiatry. 2018;175(4):359–69. doi: 10.1176/appi.ajp.2017.17010100 29145754 PMC6546164

[58] Ren X-F, Wu S-H, Zhou H, Lv L-B, Qiu Z-L, Feng X-L, et al. Maternal separation induces autism spectrum disorder in young rhesus monkeys. bioRxiv. 2022:2022.03. 17.484827.

[59] Mansouri M, Mashayekhi F, Ardalan M. Cerebellar Plasticity Changes in an Experimental Model of Autism Induced by Maternal Separation. Journal of Neurodevelopmental Cognition. 2021;4(1):9–15.

[60] Mansouri M, Pouretemad H, Roghani M, Wegener G, Ardalan M. Autistic-like behaviours and associated brain structural plasticity are modulated by oxytocin in maternally separated rats. Behavioural brain research. 2020;393:112756. doi: 10.1016/j.bbr.2020.112756 32535183

[61] Nadeem A, Ahmad SF, Al-Harbi NO, Attia SM, Bakheet SA, Alsanea S, et al. Aggravation of autism-like behavior in BTBR T+tf/J mice by environmental pollutant, di-(2-ethylhexyl) phthalate: Role of nuclear factor erythroid 2-related factor 2 and oxidative enzymes in innate immune cells and cerebellum. International Immunopharmacology. 2021;91:107323. doi: 10.1016/j.intimp.2020.107323 33385713

[62] Ahmad SF, Nadeem A, Ansari MA, Bakheet SA, Alshammari MA, Attia SM. The PPARδ agonist GW0742 restores neuroimmune function by regulating Tim-3 and Th17/Treg-related signaling in the BTBR autistic mouse model. Neurochemistry International. 2018;120:251–61. 10.1016/j.neuint.2018.09.006. 30227151

[63] Young A, Campbell E, Lynch S, Suckling J, Powis S. Aberrant NF-KappaB Expression in Autism Spectrum Condition: A Mechanism for Neuroinflammation. Frontiers in Psychiatry. 2011;2. doi: 10.3389/fpsyt.2011.00027 21629840 PMC3098713

[64] El-Ansary A, Al-Ayadhi L. Neuroinflammation in autism spectrum disorders. Journal of Neuroinflammation. 2012;9(1):265. doi: 10.1186/1742-2094-9-265 23231720 PMC3549857

[65] Roque A, Ochoa-Zarzosa A, Torner L. Maternal separation activates microglial cells and induces an inflammatory response in the hippocampus of male rat pups, independently of hypothalamic and peripheral cytokine levels. Brain, Behavior, and Immunity. 2016;55:39–48. doi: 10.1016/j.bbi.2015.09.017 26431692

[66] Levy T, Lerman B, Halpern D, Frank Y, Layton C, Zweifach J, et al. CHAMP1 disorder is associated with a complex neurobehavioral phenotype including autism, ADHD, repetitive behaviors and sensory symptoms. Human Molecular Genetics. 2022;31(15):2582–94. doi: 10.1093/hmg/ddac018 35271727 PMC9396938

[67] Mintál K, Tóth A, Hormay E, Kovács A, László K, Bufa A, et al. Novel probiotic treatment of autism spectrum disorder associated social behavioral symptoms in two rodent models. Scientific Reports. 2022;12(1):5399. doi: 10.1038/s41598-022-09350-2 35354898 PMC8967893

[68] Jenkinson R, Milne E, Thompson A. The relationship between intolerance of uncertainty and anxiety in autism: A systematic literature review and meta-analysis. Autism. 2020;24(8):1933–44. doi: 10.1177/1362361320932437 32564625 PMC7539603

[69] Desaunay P, Briant AR, Bowler DM, Ring M, Gérardin P, Baleyte J-M, et al. Memory in autism spectrum disorder: A meta-analysis of experimental studies. Psychological Bulletin. 2020;146(5):377. doi: 10.1037/bul0000225 32191044

[70] Westmark CJ, Filon MJ, Maina P, Steinberg LI, Ikonomidou C, Westmark PR. Effects of soy-based infant formula on weight gain and neurodevelopment in an autism mouse model. Cells. 2022;11(8):1350. doi: 10.3390/cells11081350 35456030 PMC9025435

[71] Rein B, Yan Z, Wang ZJ. Diminished social interaction incentive contributes to social deficits in mouse models of autism spectrum disorder. Genes, Brain and Behavior. 2020;19(1):e12610. doi: 10.1111/gbb.12610 31602784

[72] Qi C, Chen A, Mao H, Hu E, Ge J, Ma G, et al. Excitatory and Inhibitory Synaptic Imbalance Caused by Brain-Derived Neurotrophic Factor Deficits During Development in a Valproic Acid Mouse Model of Autism. Frontiers in molecular neuroscience. 2022;15. 10.3389/fnmol.2022.860275 PMC9019547 35465089

[73] Eissa N, Venkatachalam K, Jayaprakash P, Falkenstein M, Dubiel M, Frank A, et al. The multi-targeting ligand ST-2223 with histamine H3 receptor and dopamine D2/D3 receptor antagonist properties mitigates autism-like repetitive behaviors and brain oxidative stress in mice. International journal of molecular sciences. 2021;22(4):1947. doi: 10.3390/ijms22041947 33669336 PMC7920280

[74] Rein B, Ma K, Yan Z. A standardized social preference protocol for measuring social deficits in mouse models of autism. Nature protocols. 2020;15(10):3464–77. doi: 10.1038/s41596-020-0382-9 32895524 PMC8103520

[75] Colasanti M, Suzuki H. The dual personality of NO. Trends in Pharmacological Sciences. 2000;21(7):249–52. doi: 10.1016/s0165-6147(00)01499-1 10979862

[76] Tripathi MK, Kartawy M, Ginzburg S, Amal H. Arsenic alters nitric oxide signaling similar to autism spectrum disorder and Alzheimer’s disease-associated mutations. Translational Psychiatry. 2022;12(1):127. doi: 10.1038/s41398-022-01890-5 35351881 PMC8964747

[77] Caballano-Infantes E, Terron-Bautista J, Beltrán-Povea A, Cahuana GM, Soria B, Nabil H, et al. Regulation of mitochondrial function and endoplasmic reticulum stress by nitric oxide in pluripotent stem cells. World J Stem Cells. 2017;9(2):26–36. doi: 10.4252/wjsc.v9.i2.26 ; PubMed Central PMCID: PMC5329687. 28289506 PMC5329687

[78] Brown GC, Borutaite V. Nitric Oxide, Mitochondria, and Cell Death. IUBMB Life. 2001;52(3–5):189–95. doi: 10.1080/15216540152845993 11798032

[79] Hess DT, Matsumoto A, Kim S-O, Marshall HE, Stamler JS. Protein S-nitrosylation: purview and parameters. Nature Reviews Molecular Cell Biology. 2005;6(2):150–66. doi: 10.1038/nrm1569 15688001

[80] Tripathi MK, Kartawy M, Amal H. The role of nitric oxide in brain disorders: Autism spectrum disorder and other psychiatric, neurological, and neurodegenerative disorders. Redox Biology. 2020;34:101567. doi: 10.1016/j.redox.2020.101567 32464501 PMC7256645

[81] Alqahtani F, Assiri MA, Mohany M, Imran I, Javaid S, Rasool MF, et al. Coadministration of Ketamine and Perampanel Improves Behavioral Function and Reduces Inflammation in Acute Traumatic Brain Injury Mouse Model. BioMed Research International. 2020;2020:3193725. doi: 10.1155/2020/3193725 33381547 PMC7749776

[82] Burket JA, Benson AD, Tang AH, Deutsch SI. NMDA receptor activation regulates sociability by its effect on mTOR signaling activity. Progress in Neuro-Psychopharmacology and Biological Psychiatry. 2015;60:60–5. doi: 10.1016/j.pnpbp.2015.02.009 25703582 PMC5549784

[83] Won H, Lee H-R, Gee HY, Mah W, Kim J-I, Lee J, et al. Autistic-like social behaviour in Shank2-mutant mice improved by restoring NMDA receptor function. Nature. 2012;486(7402):261–5. doi: 10.1038/nature11208 22699620

[84] Anjomshoa M, Boroujeni SN, Ghasemi S, Lorigooini Z, Amiri A, Balali-dehkordi S, et al. Rutin via Increase in the CA3 Diameter of the Hippocampus Exerted Antidepressant-Like Effect in Mouse Model of Maternal Separation Stress: Possible Involvement of NMDA Receptors. Behavioural Neurology. 2020;2020:4813616. doi: 10.1155/2020/4813616 32587637 PMC7296444

[85] Sonali S, Ray B, Ahmed Tousif H, Rathipriya AG, Sunanda T, Mahalakshmi AM, et al. Mechanistic Insights into the Link between Gut Dysbiosis and Major Depression: An Extensive Review. Cells. 2022;11(8):1362. doi: 10.3390/cells11081362 35456041 PMC9030021

